# Training non-specialists in teaching recovery techniques (TRT) to help traumatised children in humanitarian settings: a qualitative analysis of experiences gained from 20 years of practice

**DOI:** 10.1186/s12939-023-01999-z

**Published:** 2023-09-11

**Authors:** Unni Marie Heltne, Anna Sarkadi, Lars Lien, Ragnhild Dybdahl

**Affiliations:** 1 Centre for Crisis Psychology, University of Bergen, Bergen, Norway; 2 Uppsala University, Uppsala, Sweden; 3 National Competence Services for Concurrent Addiction and Mental Disorders, Brummundal, Norway; 4 Norwegian Institute of Public Health, Oslo, Norway

**Keywords:** Psychosocial support in emergencies, Task-sharing, Trauma reactions, Group interventions, Children and adolescents, Global mental health

## Abstract

**Background:**

Although several manuals and guidelines have been developed to enhance the quality of task-sharing interventions, it remains challenging to train non-professional personnel in mental health and psychosocial support. Practitioners must translate general recommendations into practical applications to make them relevant in demanding contexts. However, limited research exists on practical experiences with standardised programmes in the field.

**Aim:**

To explore how experiences gained from the training of first-line health providers in a low-threshold intervention for children with trauma symptoms may guide implementation of such interventions in local communities in low-income countries. Method: We summarise 20 years of experience in the training of first-line health providers, teachers, and school counsellors in providing a low-threshold intervention. The intervention is called teaching recovery techniques (TRT), developed by the Children and War Foundation (CAW). Field notes containing notes from trainings and oral, informal feedback from participants are analysed using thematic analysis, a method well-suited for identifying, analysing, and reporting patterns, or themes, within data in qualitative research.

**Findings:**

The analysis showed three main themes/ challenges: (1) Who can conduct the TRT intervention? (2) What form and content should the training take? (3) How can the intervention be used in a responsible way to follow the ‘do no harm’ principle? We discuss the implications of the findings for trainings in scalable interventions and further research.

## Introduction

Millions of children are affected by complex emergencies, such as natural disasters, armed conflict, extreme criminal violence, and displacement in contexts of poverty and fragility. It is well-documented that war and disasters increase the risk of family violence and child maltreatment [1, 2]. All the above increase the risk of maladaptive psychological and biological development, and reduced learning capacity and social functioning. Although research has documented the resilience of children in response to potential traumatic experiences [3], traumatic experiences put children at risk of both psychological and physical health challenges, both at the time and later in life [4–6].

There is collective agreement that children and adolescents should have access to psychosocial support during and after emergencies [7]. The term ‘mental health and psychosocial support’ refers to a wide spectrum of support aimed at both protecting and promoting psychosocial functioning and wellbeing [8]. The need for psychosocial support after emergencies is highest in low and middle-income countries where there is a shortage of mental health services and health service providers. What is called the *mental health treatment gap* poses an enormous challenge globally, whereby it is estimated that fewer than 20% of those in need of mental health support receive good care [8, 9].

Community-based services and the integration of mental health in primary care are among the main strategies to reduce the treatment gap [9]. Moreover, both in emergencies and non-emergencies, care for common mental health conditions should be scaled up using a task-sharing approach in which evidence-based care is expanded to also be offered by first-line health workers and others in the local community [9]. Task-sharing (also called task -shifting) is used in a variety of settings and is described as a process whereby specific tasks are moved from specialists to health workers or others with shorter training and fewer qualifications in order to make more efficient use of existing human resources and to scale up service delivery with support from specialists [10].

Several guidelines and manuals suggest the form and content for such support [11], such as *Skills for Psychological Recovery: Field Operations Guide IASC Guidelines on Mental Health and Psychosocial Support in Emergency Settings* [12, 13] and *The mhGAP Intervention Guide Module* [14, 15]. Although the guidelines might differ in form and focus, there seems to be broad agreement on certain core principles, including ‘do no harm’, human rights and integrated services. There is also agreement that the aim of the interventions should be to protect the mental health of disaster survivors and teach skills to promote recovery and should not necessarily entail the traditional treatment of mental illness. The guidelines also include directions on what might promote the multidimensional integration of services. In emergencies, a phased approach is recommended, with different levels of intervention, from psychosocial considerations in basic services and psychological first aid that concerns all, to the more targeted intervention for groups at particular risk, and to specialised services for people in need of medication, therapy or other specialised treatment [12]. The interventions should be applicable and practical in field settings, appropriate for developmental levels across the lifespan, and be culturally informed [16]. These general intervention characteristics have also been shown to be effective in systematic reviews focusing on interventions, both for children and adults after emergencies and as part of a task-sharing strategy [17–20].

Personnel conducting psychosocial interventions as part of task-sharing are often referred to as non-specialist personnel (NSP) [10]. In addition to community health workers, this could also include persons with lived experience from the actual condition with no formal health-care role who typically belong to the same community as the beneficiary population. Other NSP groups include teachers, school counsellors and social workers. The selection of NSP is typically based on the goal of utilising local, affordable and sustainable resources [20]. Mental health specialists have diverse roles related to building competency, maintaining quality, assuring safety and (as researchers) evaluating psychological treatments [21].

Although there are several guidelines and manuals containing principles for interventions, the training of NSP in mental health and psychosocial support (MHPSS) after crises and disasters is demanding and challenging. Practitioners will have to translate general recommendations into practice, to make them relevant in a demanding context. There seems to be limited research on practical experiences with such standardised programmes in the field [22].

The aim of this paper is to explore how experiences gained from the training of first-line health providers in a low-threshold intervention for children with trauma symptoms may guide implementation of such interventions in local communities in low-income countries. We summarise 20 years of experience in the training of first-line health providers (and, on some occasions, specialists), teachers and school counsellors in providing a low-threshold intervention. The method is called teaching recovery techniques (TRT) and was developed by the Children and War Foundation (CAW) [4].

### Description of the teaching recovery techniques (TRT) intervention

TRT builds on principles of cognitive and behavioural therapies and has many elements in common with trauma-focused Cognitive Behavioral Therapy (CBT) [23]. The intervention is designed to be used in areas with low coverage of mental health specialists, mainly in low- and middle-income countries (LMICs). The intervention is intended to be delivered by non-specialists after structured training over the course of three days. On completion of training, the trainees should be able to run groups of 10–15 children. After running 1–2 groups themselves, they should be able to train others in their local community, so that the intervention can spread and reach many children over a short period of time [4]. The method has been used in many countries and has been evaluated in different contexts [24–26], although more rigorous studies are needed [27]. Over a period of years and especially more recently, the method has also been found to be useful for refugee children and adolescents in high-income countries, such as the United Kingdom [28], Sweden [25] and Norway [29], and also for adolescents exposed to domestic violence, neglect and abuse living in secure accommodation (custody) in Scotland [30]. A recent systematic review of post-traumatic interventions for children in the Middle East (including TRT) [13] concluded that teaching recovery techniques showed promising indications of reductions in PTSD symptoms, but that further evaluation and long-term follow-up studies are needed.

### Training of future group leaders and trainers of TRT

Usually, the training of new group leaders (NSP) takes place over 2.5 to 3 days and consists of a detailed description of the programme and its background and techniques. The training serves to achieve two aims: to prepare the participants to independently run groups for children, and to implement a ‘train the trainer’ concept – i.e., to prepare them to train new group leaders in their area. An example of a schedule for the training of trainers is presented in Table 1.

**Table 1:** Example of schedule for training the trainers

Day 1
Presentation
Expectations and wishes of the participants
Introduction of the training aims and schedule
Effect of trauma on children
Introduction to the Children and War/Children and Disaster Manuals
Break
Group work with children: Introduction and overview
Normalising reactions (session 1)
Recovery techniques for intrusive memories (session 2)
Dual attention task
Working on nightmares
Questions and practical exercises
**Day 2**
Summary of day one: Questions and clarifications
Principles for teaching children relaxation and arousal reduction (session 3)
Practical exercises
Break
Recovery techniques for behavioural avoidance (session 4)
Practical exercise
Recovery technique for cognitive avoidance (session 5)
Time for questions and discussion of local cases
**Day 3**
Summary of day two: Questions and clarifications
Parallel groups for parents and carers, how to inform and cooperate with parents, carers and teachers
Local implementation and evaluation
Final summary and conclusion of the training

The training is conducted in a meta-cognitive way, by including strategies on how to deliver the material, both as group leaders of the children’s groups and as teachers of the intervention to new group leaders, simultaneously. The training itself serves as a model for how to teach the intervention to others.

The trainings follow a structured pattern over three days. First, the theme is introduced in a lecture, then the participants practice the methods demonstrated, both in groups, and in pairs. The trainings follow the same structure as the group with children will follow. First there is a short introduction/mini-lecture explaining the theme of the session and explaining the rationale for the methods that are to be introduced. Then there is a demonstration of the methods to be teached in the session, followed by practical tasks for the participants to enable them to use the methods – to understand the rationale for using them, and to enable them to explain and demonstrate them to the children in the groups. The task can be in the form of exercises for pairs, in the form of individual exercises, or in the form of role plays where two participants are given the task of being group leaders and the rest play the role of children they know or have met in their work situation. The task could be as follows:

Example of role play:

*Two of the participants act as group leaders for the first session of the children’s group.*

*They will open the session, welcome the children, explain the aims of the group and establish the group rules.*

*The rest of the participants act as children.*

Example of a practical task:

*One act as the group leader and one is a child.*

*The adult group leader helps the child to make a list of reminders of the traumatic experience.*

## Method

### Aim, design and setting of the study

The aim of this study is to explore how experiences gained from the training of first-line health providers in a low-threshold intervention for children with trauma symptoms may guide implementation of such interventions in local communities in low-income countries.

This is an explorative study that uses qualitative material gathered through 20 years, to analyse experiences. The setting of the study is 29 different training sites in 19 countries, most of which are LMICs in Asia, Africa, and the Middle East. The trainings have taken place during or after wars and after major disasters, such as earthquakes or flooding. Trainings have also been undertaken after major accidents, such as transportation accidents or fires.

### Material

The material consists of two of the authors’ personal field notes made during and after trainings, and written records of participants’ questions and comments (not gathered through feed back questionaires) that were also made during and after trainings. This material has been supplemented with notes from informal discussions with co-trainers, and informal written feedback (not systematic evaluations of answers to questionnaires) from participants of the trainings. The informal material has been transcribed and organised by the first author and discussed with the other authors during the analytical process. Some of the field notes are rather detailed and describe experiences and reflections, whilst other notes are just in the form of quotes or single key words. The material was gathered from 2005 to 2023. Examples of the material and its content are showed in Table 2 below.

**Table 2:** Example of material and content

Type of material	Example of information
Trainer’s reflection in field note during the training	The purpose of the introduction needs to be more detailed and clearer
	The different terms such as *reminders* and *intrusion* need to be explained more clearly
	We need to use more demonstrations and practical exercises to explain the point of gradual exposure
Recorded question from participants	“How do we explain trauma reaction to the children?”
	“How do we maintain order in the children’s group without using harsh discipline?”
	“How do we get the children to talk in the group?”
Discussed among the trainers and written down as an example to be used in future planning and trainings	Example of challenging situations with reactions from participants who are traumatised

The material also included notes from oral evaluations with participants at the end of the training, where the participants gave feedback on what had been useful in the trainings and what could be improved. This feedback was also written down as part of the trainer’s field notes. Some of the material was used in its handwritten form, whilst material that was difficult to read was transcribed using a PC. The material thus consisted of 95 pages of notes of varying quality and length.

### Analysis

The material was analysed using thematic analysis, as described by Braun and Clarke [31, 32]. This method is well-suited for identifying, analysing, and reporting patterns, or themes, within data. It is a flexible and useful research tool and can be used for many kinds of qualitative data. Thematic analysis can be conducted in either an inductive or a theory-driven way. Here the analysis is done on the basis of a explorative, constructivistic approach. The explorative approach is used to explore new areas and generate ideas, while the constructivist approach focuses on understanding subjective experiences and meanings from the participants’ perspectives. We have tried to code the data without using any preexisting framework, but as all notes and observations were made and recorded by the authors, it is also driven by the authors’ preconceptions, experiences, and expectations. The six steps of the analysis are: (1) familiarising with the data by reading and re-reading the material to obtain an overview, (2) generating initial codes that cover what the data are about as broadly as possible, (3) searching for themes that contain what the codes describe, and steps 4 and 5, which involve refining, adjusting and relating the themes to the whole material. After the final themes have been established, text extracts or examples are chosen to illustrate the themes, step 6.

In this study, the first author read through all the material, and formulated codes that encompassed most of the material. After this, the material was reread by one of the other authors, and independent codes were made. A process of triangulation was used to evaluate the trustworthiness of the analysis. The two code sets were compared to reach a common set of codes. When comparing the codes, a high degree of similarity between the sets of codes was found. The same process was repeated to generate the initial themes. Two of the authors constructed separate sets of themes, which were then compared, and agreement reached upon the final themes.

The material was then reread with the purpose of adjusting and developing the main themes further. The process resulted in three main themes with sub-themes, with each theme describing recurrent challenges (see Table 3). Descriptions of concrete situations from trainings were selected to illustrate some of the themes.

**Table 3:** Example of the analysis

Phase	Step 1 Familiarising with the material	Step 2 Generating initial codes	Step 3 Searching for themes	Step 4 Reviewing themes	Step 5 Refining and naming the themes
Responsible	First author	Discussion between authors	Discussion between authors	Two of the authors	
Step		Initiating codes across the material. Collecting quotes and notes related to each code.	Gathering codes under each theme. Creating a thematic map.	Ensuring that the theme works in relation to the coded and entire data set. Generating a thematic map.	Ongoing analysis to refine themes and the overall story told by the analysis.
Example of content: Notes of questions asked during the trainings. Notes of our own reflections and observations during and after trainings.	Questions and extracts from the notes: “Participants listing a lot of reactions, not all of them related to trauma.” “Requests for more practical exercises.” “Participants asking about common reactions to trauma.” “Observed need for more clarification regarding normalisation.” “Need for clarification of concepts such as intrusion and avoidance.” “NGO asking who could be recruited for the trainings, and what background knowledge they need.” “How do we keep order in the group?” “How do we get children to participate?” “What do we do with children who do not want to talk?” “What if a child tells us about abuse at home?”	How to create a nurturing group climate. Need for concrete knowledge of reactions to potential traumatic experiences. Need for concrete knowledge of methods to reduce these reactions. Who can perform the interventions? Will the intervention work in our context?	Preliminary themes: Format and content of the trainings. How to use the method in a responsible way. Recruitment and selection of group leaders (trainees) NSP. Contextual factors	Overarching themes: 1)Who can do it, who to recruit, selection of NSP. (What if the trainees are traumatised?) 2) How to teach/train a.) Form (interactive, use of roleplays, group tasks, feedback). b.) Content (concrete methods to reduce reactions, handling of challenging situations in the children’s groups and in trainings). 3) Do no harm a.) Balancing fidelity with flexibility (adapting to the context). b.) Ensuring children’s safety and security. c.) How to integrate intervention with other interventions. d.) How to implement with inadequate resources.	

### Ethical considerations

In the material no personal information has been registered. We have protected anonymity by not gathering or describing any personal or identifying information. The described situations used to exemplify the findings have been modified and sometimes constructed by the condensation of several experiences into one example. They are situations from trainings, but contain no identifiable information.

## Findings

The analysis showed three main themes/challenges: (1) Who can conduct the TRT intervention? (2) What form and content should the training take? and (3) How could the intervention be used in a responsible way to use the intervention in a responsible way to follow the “‘do no harm’” principle? All the main themes could be divided into several sub themes.

### Who can be trained to use the TRT intervention?

This challenge/theme is generated from questions frequently asked by local Non Govermental Organisation (NGO)s or official organisations when starting to plan a training. Examples of questions asked include: “*What background should the participant have?*”, “*Do the trainings require any particular education?*”, “*Could the participants be included even if they also had trauma reactions to what has happened?*”, “*Can experience of working with children compensate for a lack of education?*” and “*Can people other than teachers or nurses participate?*” These questions were repeated in almost every training.

#### Background, education and knowledge of the participants

The comments and notes indicated that, to be trained as a group leader for TRT, it is an advantage for NSP to have basic knowledge of child development or experience of interacting with children. Also, the participants should be able to reflect upon how adults’ behaviour will affect children’s behaviour and emotions. Notes both during and after trainings, and notes from discussions after trainings, indicated that it is easier for trainees with some post-high-school education to understand the basic concepts and theoretical background of the method. In some areas, it is difficult to find enough participants with such qualifications, but the findings also indicated that broad experience of working and interacting with children can compensate for lower levels of education.

#### Personal skills

Basic interactional competencies such as communication skills and genuineness, the ability to show respect, and the ability to foster positive coping strategies and a sense of optimism are important, but these are quite hard to evaluate in a selection process before the training starts.

#### Participants who are themselves traumatised

A recurrent comment was that the local adults who are trained to conduct training may themselves be traumatised and experience distress during their training. Exposure to the trauma reactions and experiences of children could trigger their own reactions. This does not necessarily make them unsuitable for the task, but it must be addressed during the training. Several comments underline the importance of normalising reactions and applying trauma knowledge to increase understanding, and to emphasise skills to reduce and control the reactions to ensure that they do not interfere with the leading of the group and the guiding of the children. It is important to have a plan for referral to supervision or treatment for participants who express strong and uncontrollable reactions. Sometimes it is possible to use participants’ reactions to illustrate for the whole group how expressions of distress could be handled in the group with children.

*In a training of group leaders for TRT, one of the participants burst into tears when the group was asked to brainstorm traumatic reminders. She was reminded of a situation when she had been chased by hostile soldiers. The leader of the training explained to the whole group that crying was exactly one of the reactions that could be expected when talking about reminders. When the participant had regained control, the instructor thanked her for giving the group an opportunity to learn how to manage a situation when a child starts to cry in the group.*

One finding was that it can increase the motivation of the trainee if he or she experiences the effect of the methods:

*In a training, one of the participants experienced very vivid flashbacks while participating in a roleplay illustration of how to help children to gain control over such reactions. The method demonstrated did not seem to be sufficient to reduce the participant’s flashback. After the session, she was instructed in using several relaxing techniques, and she then tried out one of the techniques to reduce flashbacks together with the main trainer. This time, she experienced immediate relief. She reported back the next day feeling much more in control and was very motivated to teach the technique to children who suffered from the same symptoms.*

### Form and content of the training

#### Creating a facilitating and supportive group climate

The following questions from the participants during the trainings (see examples in Table 2) and the fieldnotes of the author (see also Table 2) were the basis for this theme: “*How can we explain the purpose of the group?*”, “*How do we keep order in the group?*”, “*How do we get children to participate?*”, “*What do we do when children do not want to talk? How can we create a good group climate?*”

The theme was also based on the authors’ fieldnotes: “*Participants asking about common reactions to trauma*”, “*Observed need for more clarification regarding normalisation*”, “*Need for clarification of concepts such as intrusion and avoidance*”. Notes from discussions of the value of talking about reactions and difficult experiences were also part of the material for this theme.

The findings based on the field notes showed that the training of NSP as group leaders for TRT should be interactive and practical – e.g., teaching the methods by showing and practising. The more practical activities that are integrated in the training, the better.

The importance of creating a facilitating and nurturing group climate was stated both in questions from participants and in field notes. Also, it was underlined that the groups must be led in a supportive and accepting style, and the group leaders must not use harsh discipline or any form of punishment. Adults who used physical violence against children would not be suitable to lead the groups.

In several informal evaluations of the training of TRT, the trainees requested more opportunities to practise the intervention and to get feedback. The participants are encouraged to continue practising roleplay, where they act as group leaders and try out giving information, interacting with the children, and demonstrating the various techniques after the initial training. They are also encouraged to establish peer-to-peer supervision to support each other.

#### Direct and open communication with children

One challenge that NSP experienced was related to direct and honest communication with children who participate in the intervention groups. This often became apparent when the trainees were asked to demonstrate how they would explain the content and the purpose of the group to the children. It seems that many participants tended to play down the hard and challenging parts of the group intervention, often with the purpose of motivating the children to participate. They could describe the main purpose of the group intervention as “a place where we should have fun, a place where we can play and enjoy ourselves”. However, it is important that the children have realistic expectations of what to expect from the group participation and understand the aim of the group. It was frequently noted that it was necessary to rehearse how to describe the purpose of the group to children. It was noted that, although the manual suggested ways of phrasing this, the proposed formulations would not necessarily suit every language or local form of expression, but it was stressed that, whichever words the group leaders choose, they should make sure that the meaning was clear: the purpose of the group is to learn and practise methods for coping with bad memories and fears after experiencing something very frightening.

#### How to understand, explain and normalise trauma reactions

This was a recurrent question in the field notes, as well as in recorded questions from participants. In the training of some cultural groups however, talking about trauma reactions is considered inappropriate or even harmful, and it is believed that focusing on painful reactions could reduce the children’s coping capacity and resilience. The same approach could be applied in the case of encouraging children to talk about threatening experiences. In some contexts, talking about frightening experiences is believed to make the reactions worse, and should be avoided; instead, children should be helped to forget, not be reminded of, the frightening situations. Balancing the adaptation to local culture with the evidence-based manual is also a challenge, and we have found that this is an issue that must be discussed with the trainees.

Other recurrent questions regarding the content of the trainings are how to explain central terms such as reminders, intrusions and avoidance, and how to demonstrate and teach children the self-help methods, especially the principle of gradual exposure when children are avoiding reminders.

### Using the intervention in a responsible way to follow the ‘do no harm’ principle

This theme was also based on frequent questions from the participants and reflections in the fieldnotes: “*Can we change details in the manual to suit our context better*”, “*Can we use more locally acceptable ways to describe the reactions we talk about?*”, “*Do we have to do all the sessions in the way the manual describes them?*” and “*Can we omit parts we do not think are suitable in our context*?”

The author notes regarding this were included: “*Important to stress that the schedule of the sessions should be followed*”, “*Content can be changed as long as the initial meaning is understood and kept*” and “*New theme or methods should not be added to the initial programme.*”

#### Balancing flexibility and fidelity

Group leaders were always recruited from the local population to ensure that the programme is culturally acceptable and relevant in the local context. In the notes, it was underlined that the training should ensure that the method and the training material, with its content and concepts, were appropriate, suitable and understandable not only for the children in the groups, but also for the future group leaders. Sometimes, it was necessary to spend quite a lot of time on this issue. Some languages may lack concepts such as flashbacks and intrusion, and in other settings the story used to exemplify common reactions to trauma needed to be adapted to the local context.

*While training social workers in a crime-ridden area in South America, the participants found that the story of a regular war scene was not recognisable enough for the children in their context and modified it to a situation involving violence between criminal groups.*

In other situations, words such as trauma reactions and post-traumatic stress needed to be changed and modified into other words, because the local words for trauma and traumatisation were related to psychiatric illness and were stigmatising. Elsewhere, these words might be unknown and have no meaning.

Sometimes the group leaders had problems handling the content of the children’s experiences, as well as their own reactions to these narratives. When this happens, the children could perceive the adults’ reactions as a signal that their stories were too bad to be told and believed that they should not share them. Handling such reactions requires confident and experienced adults, and this is the main reason why the focus on the children’s individual stories is delayed until both children and group leaders have acquired some tools for regaining control.

Participants often wanted to discuss what to do if one or more children started to talk about frightening and traumatic experiences at the first meeting. In the trainings it was found useful to give the participants some guidance on how to handle this in ways that did not discourage the children from telling their stories, while still making the situation manageable for both the group leaders and the other children in the group.

*In a group of adolescents, two of the boys started to talk about killings and abuse by soldiers that they had witnessed when trying to escape from their occupied hometown. Both boys were very emotional, and the group leaders feared they would not be able to calm the boys down. They were also concerned that their stories would upset the other participants and trigger reactions that would overwhelm them. They confirmed to the boys in the group that their stories were exactly why they were meeting, and why they would continue to meet for the next four sessions. They would be invited to talk and think about their stories on several occasions, but first they wanted both boys to tell their stories to the adults only. They then asked the group if it would be OK to have a break and invited each boy to talk about their stories with the group leaders. In monitoring the boys’ reactions to telling their stories, they tried to evaluate how upsetting the situation was for them. After telling the story, they were then individually informed of some of the self-help methods they would learn in the group, as well as how they could handle reactions to and thoughts about the event after the group session. In the plenary group after the break, they were praised for their courage in attending the group and were reassured that they would have the opportunity to tell the group more about their experiences in the later sessions. The other group participants were very supportive and confirmed the group leader’s praise. The whole group was then taught self-calming and distraction techniques.*

One finding was that the importance of following the manual and avoiding changes or skipping content should not only be stressed, but also discussed and explained. For example, in the two last sessions (sessions 4 and 5), where graded exposure is presented to and practised with the children, the group leaders would sometimes suggest skipping or changing some of the content. There were several reasons for this: the NSP might find it difficult to elicit difficult reactions from children and fail to see the long-term benefit of exposure training; children are often themselves not initially very keen on exposure training, and leaders may then encounter negative reactions from the children, making them wary of the content; and finally, these two sessions are at the end of the training, and group leaders intuitively want to leave the children in a stable and happier state. Therefore, it seemed especially important to discuss the challenges associated with sticking too closely to the manual with group leaders, both when conducting groups and when training new group leaders. The results point to the importance of offering participants options to solve such situations, for example by organising a follow-up, farewell session two weeks after the fifth session to meet the children and see how they were doing, or by sharing stories in the training from children who have benefitted from graded exposure despite initial resistance to the method.

#### Ensuring children’s safety and security

In the material, there were several records of participants talking about children who were abused or could be in danger of being abused. The question “*What can we do if a child talks about being abused at home?*” was asked rather frequently. The results based on the field notes showed the necessity of discussing whether the encouragement to talk about traumatic experiences could imply a risk of the children being punished for such openness at home. This is illustrated by the following field note examples:

*“Must discuss whether participation in the group implies any risk of sanctions for the children.”*

*“The issue of a safety plan for children at risk of abuse must be focused.”*

This was described as a very challenging theme, and it was not always possible to find a satisfactory solution to how to handle worries of child abuse or neglect in the TRT trainings. This depended on available resources, attitudes and customs for child protection, and the knowledge of the harmful effect of child abuse. In some trainings, this theme was addressed and discussed at length. The importance of having local participants raising the issue and leading the discussion was emphasised. The experiences underline the need to raise the awareness of the fact that participation in the groups and the encourage talking about traumatic experiences at home, could sometimes put children to be at risk of abuse. Ways to to avoid this should always be discussed and implemented. In some of the feed back, the participants in the TRT training expressed concerns that even for children to reveale their names in the group could put them and their families at risk.

#### How to integrate TRT with other interventions

Examples of recorded questions related to this theme include: “*What is the difference between this intervention and emotional first aid?*”, “*Can this intervention be used as part of a child-friendly space?*” and “*Could this intervention be used in the emergency situation?*”

The field note contains a comment that sometimes there is a lot of confusion concerning when to use the TRT intervention compared to other interventions. In an emergency situation there might have been a lot of trainings by different NGOs, presenting a wide spectre of interventions, but no presentation of a useful structure showing the relationship between the methods and their different aims and timing. This challenge therefore stood out as another sub theme.

#### How to implement the intervention with inadequate resources

Many questions during the training were concerned with the lack of material and human resources, and how to implement the method in such situations. Questions related to how to get any supervision and support, how to find the time to run the groups for children, and how to acquire a minimum of resources, such as paper and pencils, or some food for the participating children, were recurrent throughout the material. Similarly, the challenges of acquiring supervision and organisational support were also raised.

## Discussion

The aim of this study was to explore how experiences gained from the training of first-line health providers in a low-threshold intervention for children with trauma symptoms may guide implementation of such interventions in local communities in low-income countries. Field notes and records of participants’ questions gathered over the past 20 years have been analysed using the method of thematic analysis (Braun and Clark). The analysis resulted in three main themes/challenges: (1) Who can conduct the TRT intervention? (2) What form and content should the training take? (3) How can the intervention be used in a responsible way to follow the ‘do no harm’ principle? In the following, we will discuss the challenges identified in the analysis, considering current literature and possible ways to handle them.

### Who can conduct the TRT intervention

#### Selection of group leaders

The selection of future group leaders is a keyissue in all literature on task-sharing [33, 34]. Some qualities are general and common [35], whereas others are specific to the intervention at hand. Familiarity with the context in which the psychological intervention will be delivered should be mandatory [22].

Although it is easier to conduct any kind of training with participants with similar backgrounds and experience, attempting to assemble a homogeneous group can be unrealistic. Participants with quite different backgrounds might provide diversity, which is valuable for input both to participants and trainers. Teachers have different experiences to those of nurses, and social workers or counsellors will have different experiences to those of medical doctors. Participants from rural areas might have different perspectives than those from larger towns.

It might also be unrealistic to expect that all participants in a training will be able to advance to become group leaders after the training, and the organising partner may have to plan for how to select and monitor the participants’ efforts as they go on to practise their skills in running children’s groups [36].

If a group leader has previously been traumatised, this could increase the risk of secondary traumatisation induced by the children’s stories. It is therefore important to establish routines and procedures for caring for the staff, such as supervision, peer support and regular feedback [37]. Several guidelines for staff support in emergency settings have been developed [38], but unfortunately these are often poorly implemented [34]. Proper staff support is essential to prevent burnout and loss of commitment, which is a constant risk when working with traumatised populations in situations with limited access to training, support and material resources [38]. This might however be difficult to arrange in emergency situations and in situations with small or no resources. Specialist supervision might not be available at all, and there might not be any time to spend on supervision. Several group leaders lead children’s groups in addition to their regular work as teachers, nurses or other occupations. It is therefore important to spend time, both before and during the training, to discuss and plan ways of securing some kind of supervision, such as peer-to-peer supervision in pairs or groups, arranging experience meetings or online supervision.

### Form and content of the trainings

#### Different opinions on the value of talking about painful reactions and traumatic experiences

One essential part of TRT is explaining and normalising trauma reactions. This is a, such as by acknowledging the differences between the early expression of strong emotion and the regulation (calming down) of strong arousal, and by introducing a timeline for when talking or not talking might be helpful [39]. The way in which the talking is conducted also seems to be important, including a focus on creating an overview and a coherent narrative, and clearing up possible misunderstandings and misinterpretations [40, 41]. In such a context, talking about the traumatic experiences seems to be clarifying and helpful. Given the controversies both in the professional field and among people of diverse cultures, it is understandable that this issue can lead to insecurities and resistance. The resistance to talking about traumatic experiences and painful reactions must therefore be openly addressed, both in the training of group leaders and in preparatory meetings with parents/carers. This issue should also be considered in relation to the issue of integrated and holistic response and care for children in adverse circumstances. TRT and trauma support will never address all of the children’s needs or rights and will not be needed for all children. Therefore, the phased approach described in the Inter Agecy Standing Comitte (IASC ) [12] guidelines are crucial, whereby TRT and other mental health interventions are seen in the context of children’s and communities’ overall situations and provisions.

#### Creating and facilitating a nurturing and supportive group climate

To make the TRT intervention function, it is important that the group leaders will be able to create a group climate that is safe and free of sanctions, and that stimulates interaction both between the children and between the children and the group leaders. The trainings therefore emphasise that the children should be encouraged to be active from the very start of the group, and they must feel safe to express their suggestions and experiences freely. However, if they are passive or do not complete their tasks, they should not be met with sanctions, but rather be encouraged to try next time. Depending on the existing interaction pattern between adults and children in a particular culture, this can be self-evident or very challenging, and methods to achieve this are discussed with the trainees. If a non-didactic interaction with children is unfamiliar, it is necessary to illustrate and roleplay alternatives. It might also be necessary to illustrate how to maintain order and structure in the children’s group, without disciplining the children.

To create a positive and supportive group climate, the group leaders must interact with the children and interact with each other in a way that demonstrates this. For example, we often introduce each theme by having the children brainstorm suggestions and ideas. When working on the normalisation of trauma reactions, the children could be asked for examples of how we react when we are afraid, and how they think the child in the story would react after the traumatic experience.

Based on our experiences we recommend that the children should be encouraged to suggest which rules they would like to have for the group, and the children’s suggestions should all be accepted and praised (as long as there are no destructive or aggressive suggestions). If the group rules are formed in a sense of cooperation, the children will gain ownership of them, and the rules can be used as a tool to maintain the structure of the group without having to use disciplinary strategies, such as time-outs or scolding. This advice is also in accordance with general recommendations for user participation stated in intervention guidelines for psychosocial support [7]. In the same way, the interaction between the trainers can be used to exemplify cooperation, respect, and sometimes playfulness and humour. In this way, the trainers can model how to create a good group atmosphere during the trainings and explain to the trainees what they are doing – for example, when they invite the trainees to participate actively from the beginning, and when there is a non-didactic relationship.

To ensure a safe and nurturing group climate, it is also important to ensure that the group leaders are adults who do not use authoritarian or violent disciplinary methods outside the children’s groups. It might be considered self-evident that the use of violence against children is counterproductive to methods to reduce trauma reactions. However, the use of violence, physical punishment, and humiliation against children in schools and homes is common in several contexts [42, 43]. Therefore, this theme must be addressed, both before recruiting trainees and during the training, to ensure that the children are safe in the TRT group. An adult who has a history of using violence to discipline children, either as a teacher or as a carer, cannot function as a group leader in groups aimed at reducing trauma reactions. The prevention and removal of violence against children is an important task, but it is also complicated as it can be firmly embedded in the child-rearing practices in many contexts. The ability to communicate the harmful effect of violence on children’s development and wellbeing demands the use of language that is non-moralistic and non-patronising, and that at the same time avoids cultural relativism. We have found that sharing neutral facts about child development, neurological development, and the harmful effect of violence on learning capacity, memory and general functioning is a meaningful strategy that often stimulates curiosity, and an open discussion makes it possible to get the message through. This is especially effective if the provision of facts and discussions are headed by local trainers.

### How can the intervention be used in a responsible way to follow the ‘do no harm’ principle?

#### Balancing flexibility and fidelity

Ensuring fidelity is underlined as one of the factors that are important to ensure the general success of task-sharing [35]. This is a guarantee for the responsible use of the method, especially when close supervision and monitoring after the training are difficult.

The manual developed by CAW is made as simple as possible without losing core content, so that NSP with different backgrounds, competencies and skills can use it. Based on CBT and many elements that are similar to trauma-focused CBT [40, 41], the manual covers both simpler and some more complicated methods, such as gradual exposure and methods derived from eye movement desensitisation and reprocessing (EMDR) [44]. The methods are made simple and practical to facilitate understanding by people without extensive theoretical or therapeutic backgrounds and training. For this to work, it is important that the NSP follow the manual as closely as possible, and refrain from removing things or adding more advanced material. The emphasis on fidelity is similar to the *mhGAP Mental Health Programme* [45].

It is widely agreed that culturally adapted psychological interventions are associated with greater effectiveness than non-culturally adapted psychological interventions [46]. In our experience, such adaptations to culture and other contextual factors are well-suited for discussions with the trainees throughout the training to make sure that the content is meaningful and understandable for both the trainees and the children. There will often be local methods for relaxation, distraction and exposure that could be included in the programme.

The balance between securing fidelity and adapting the intervention to a cultural context can sometimes be challenging and may reduce the effect of the intervention [47]. For example, normalising reactions by talking about trauma reactions and using the story of a traumatised child as an example have sometimes been omitted from the programme because of cultural beliefs that talking about painful reactions could harm the child’s resilience. However, our impression is that this has reduced the intended effect of the intervention, although this still needs to be tested empirically. Even if there is evidence for the positive effects of psychoeducation, exposure and the use of the trauma narrative, there is still a need for further research to identify the components that are effective and that create changes both in TRT and in other similar programmes [48–52].

Following the manual also means following the sequences of the programme. The themes and techniques in the sessions follow a fixed sequence that is built on a defined rationale: the children should be taught about the nature of trauma reactions, and then taught some techniques to regulate painful emotions before they are encouraged to approach the things they avoid because it reminds them of the trauma. This could involve physical exposure or encouragement to talk about their traumatic experiences. In fact, they are not encouraged to talk about their experiences until the fifth and final session. The literature on trauma-focused CBT suggests that the use of the trauma narrative could come much earlier than when the therapist usually introduces it, and this postponement is more to do with the adult’s reluctance than the needs of the children [49]. In the case of task-sharing and the use of NPS, however, it is important to take into consideration the fact that the group leaders of TRT are not experienced therapists or trauma counsellors. They may also be teaching the method to others who do not have the same kind of background or the same amount of training as they do.

#### Awareness of risks and need for security plans and protection

In some contexts, the mere activity of talking about one’s own experience could put a child (or a group leader) at risk. In this situation, a very thorough evaluation must be made as to whether the intervention should be implemented at all. This might be the case in communities with high rates of criminal activity, or where suspicion of whistleblowing to the police, to other local authorities or to the army is severely sanctioned [26]. Such situations show the need for gathering in-depth local knowledge of the context before planning training and implementation. This will be the case before implementing any trauma-relieving or treatment method, but there is a particular risk when implementing a method whereby children are encouraged to talk about their frightening experiences to somebody else. In some of the training’s participants expressed concerns that even reveal their names in the group could put children and their families at risk. In such situation the intervention should be put on hold.

A similar situation occurs when psychosocial interventions are used for children who have been exposed to domestic violence and/or abuse. Sometimes, encouraging children to talk about their experiences of violence and abuse by parents or other adults inside or outside the family can put the children at risk of more abusive actions from adults, punishing them for telling their stories. If insufficient attention is paid towards the child’s safety in these situations, the intervention could be dangerous. In general, a lack of knowledge or routines for how to handle such high-risk situations could be a serious barrier to the implementation of evidence-based interventions in LMICs [43, 53]. The creation of routines for safety in relation to the implementation of psychosocial interventions for children must therefore be a high priority [43].

While focusing on how to adapt the material to a specific context, it is also important to address possible security issues [43]. If there is any chance that participation in the intervention could put a child at risk of violence, exclusion or other harmful consequences, the intervention should not be used. To ensure that it is used in a non-harmful, responsible way, it is also important that trainees are aware of when a child or adolescent needs more specialised and/or individual help. The group leaders should have some basic tools for handling suicidal thoughts and plans. In a low-resource setting, specialised help is not always available, which makes establishing a procedure for how to handle suicidal group participants even more important. Basic training in psychological first aid [53] may be useful for all trainees before they attend TRT training. When used in a high-income country such as Sweden, group leaders have expressed that dealing with the threat of suicide within the group is regarded as one of the biggest challenges when running groups for unaccompanied refugee minors [54].

The mhGAP programme [45] includes a module that focuses on how to both discover and handle suicidal thoughts and plans, and it is possible that this module could be combined with the TRT training. Alternatively, training in psychological first aid, where some attention is also paid to suicide prevention, should be considered. Dealing with the challenges of suicidal participants has been a specific reason for reluctance to train non-specialists to provide psychosocial support, either to traumatised people or to people with general psychological disorders such as anxiety or depression. Regarding training, it is important to spend time on planning how to support the NSP and establishing routines and methods for supervision.

#### Integrating the intervention with other resources: understanding the intervention level and when to use it

After an emergency, a diverse range of training programmes are put into use by many different agencies and NGOs. These should be coordinated by the government or delegated to one or more agencies or organisations. However, this coordination can be ineffective and sometimes lacking in overview, as coordination is often challenging, Whatever the quality of this coordination, it is important to place the intervention used in the context of other interventions and of a timeline, and within a framework of the existing infrastructure and services. Trainees have sometimes been confused by the relationships between TRT and psychological first aid [55], child-friendly spaces and other interventions for the general population, but this is relatively easy to clarify. As TRT is an intervention that targets defined trauma reactions, it could be placed in the IASC intervention pyramid [56] as targeting populations with special needs, but not as a specialised intervention. This should be made clear to participants at the beginning of the training, as should the differences between general and targeted interventions.

#### The challenge of sustainability and scaling-up in a context of scarce resources

How to facilitate the scaling-up of useful interventions once a successful training is over is almost always found to be a problem. It is well-known that many training approaches may lead to increased knowledge but not necessarily to the use and spread of the specific intervention, even among mental health specialists [56]. There can be many barriers to taking the step from using the intervention to training others [57]. In many cases, being trained in a method is not enough. There is a need for prolonged programmes on how to train the trainers and how to scale up, but this can be quite difficult in a low-resource setting. One way could be to organise local supervisors and peer supervision as part of training in the method, and to set rules for when it would be advisable for the trainees to have reached a level of competence where they could start training others. Several manuals have been developed for training the trainers [53], but the challenge is often a lack of available time and resources to set up such trainings [33, 35]. There is a need both for the further development of standardised methods and measures of competence for NSP [33] and for the demonstration of the reliability and validity of peer supervision [16].

The issue of sustaining NSP-delivered treatments and motivating NSP also needs attention [58]. The lack of resources and plans for achieving sustainability is often a key obstacle to the continuous spreading and use of interventions, as well as being a potential stressor for the NSP [53]. On the other hand, the need for the further development and evaluation of measures and methods should not prevent or postpone the use of methods that have been shown to be effective or promising. Based on our experience, we think that integrating TRT knowledge into the preservice training of health and social workers may be a fruitful approach, both to secure sustainability and to support task-sharing on a larger scale [59].

Recently there have been several attempts to develop digital intervention programmes that can be downloaded as apps on mobile phones and tablets. This is a possible approach for both the training [60] and the use of the TRT intervention [61]. The practical and standardised way in which both the trainings and the interventions are described could make such an adaption successful [62].

### Limitations

This study has several limitations. The use of field notes and other informal records that are not aimed at conducting research make the systematisation process difficult, as does the comparison of materials of very different quality. As the notes were made by the authors, the observations and the choices of examples are coloured by preconceptions, prior experiences and attitudes. It is therefore impossible to know how the same observations would have been selected and focused by an independent observer. Moreover, we do not have knowledge of other challenges that might have been raised by using an alternative method for the collection of information. For example, it is possible that systematically gathering evaluation data by means of structured interviews or a questionnaire could have given other results. However, the material has been gathered over a long period of time and in many different contexts, and therefore provides meaningful input for understanding and improving low trauma support in the context of task-sharing. Also the challenges observed here are quite in line with comparable literature in the field [10, 11, 22, 38]. The fact that certain challenges and strengths were brought up over a period of years and across contexts indicates that these are important issues that may provide meaningful understanding for the further development of task-sharing interventions, despite the methodological limitations of the current study and they can serve as examples of possible ways to handle challenges that arise when also training NSP in methods other than TRT.

## Conclusion

The implementation of a manual-based intervention used in a task-sharing approach involves several challenges. Many of these are common across interventions and contexts. Evaluations and outcome studies of TRT and similar interventions tend to focus more on the effects of the intervention on participating children, and less on the perceived challenges in training and capacity development. The current study has provided new knowledge about important topics for such training and implementation.

The systematic analysis of the experiences gathered in trainings of NSP could contribute to finding solutions to some of the challenges. The selection of NSP, the form and content of the trainings, and how to use the intervention in a responsible manner by doing no harm are especially important. The balance between simultaneously securing both fidelity with the method and sufficient flexibility to adapt to a certain cultural context is important. Securing safety and safeguarding routines for children faced with threatening conditions should always be a major consideration. It is important to be able to place the intervention within a wider framework of support in the community, and to have strategies for how to spread the intervention to as many children who are in need of the intervention as possible. At the same time, there is both a great need for research on the effect of all these elements and future method development to make interventions such as TRT more accessible.

## Data Availability

The raw material for the analysis can be accessed by contacting the first author.

## Abbreviations

CAW: Children and War Foundation
CBT: Cognitive Behavioural Therapy
EMDR: Eye Movement Desensitisation and Reprocessing
IASC: Inter-Agency Standing Committee
LMIC: Low and Middle-Income Country
MhGap: Mental Health Treatment Gap
MHPSS: Mental Health Psychosocial Support
NGO: Non-Governmental Organisation
NSP: Non- Specialist Personnel
TRT: Teaching Recovery Techniques
