# Correction: Training non-specialists in teaching recovery techniques (TRT) to help traumatised children in humanitarian settings: a qualitative analysis of experiences gained from 20 years of practice

**DOI:** 10.1186/s12939-023-02021-2

**Published:** 2023-10-11

**Authors:** Unni Marie Heltne, Anna Sarkadi, Lars Lien, Ragnhild Dybdahl

**Affiliations:** 1 Centre for Crisis Psychology, University of Bergen, Bergen, Norway; 2 Uppsala University, Uppsala, Sweden; 3 National Competence Services for Concurrent Addiction and Mental Disorders, Brummundal, Norway; 4 Norwegian Institute of Public Health, Oslo, Norway

**International Journal for Equity in Health (2023) 22:187**

10.1186/s12939-023-01999-z

After publication of this article [1], the authors reported that in the Introduction, under the heading ‘Description of the teaching recovery techniques (TRT) intervention’, first sentence, ‘Congnitive Behabioral Theraphy (CBT)’ should have read ‘Cognitive Behavioral Therapy (CBT)’.

The original article [1] has been corrected.
